# Supplementary material for: Dairy-Derived Emulsifiers in Infant Formula Show Marginal Effects on the Plasma Lipid Profile and Brain Structure in Preterm Piglets Relative to Soy Lecithin
Source: Nutrients. 2021 Feb 24;13(3):718. doi: 10.3390/nu13030718 (PMC7996312; doi:10.3390/nu13030718)
Supplement: Supplementary file 1 [file nutrients-13-00718-s001.zip › nutrients-1104973-supplementary/nutrients-1104973-supplementary/SupplementaryMaterial-NutrientsProofreading.docx]

**Supplementary material**

| Compound | Gastric step concentrations | | Intestinal step concentrations | |
| --- | --- | --- | --- | --- |
|  | Medium (mM) | Final assay (mM) | Medium (mM) | Final assay (mM) |
| Sodium chloride | 34.1 | 3.0 | 110.9 | 47.4 |
| TRIS | 23.1 | 2.0 | 2.0 | 2.0 |
| Maleic acid | 23.1 | 2.0 | 2.0 | 2.0 |
| Calcium chloride | 64.8 | 4.4 | - | 2.8 |
| Sodium taurocholate | - | - | 2.4 | 1.0 |
| Phospholipid | - | - | 0.5 | 0.2 |

**Table S1.** Concentration of simulated gastric and intestinal media used for two-step pediatric in vitro lipolysis model

**Table S2.** Organ weights relative to body weight on day 19 in WPC-A-EV, WPC-PL and SL groups (mean ± sd)

|  | **Relative organ weight (g/kg)** | | |  |
| --- | --- | --- | --- | --- |
| **Organ** | **WPC-A-EV (*n*=22)** | **WPC-PL (*n*=19)** | **SL (*n*=19)** | ***p*** |
| Small intestinal length (cm) | 287 ± 55.8 | 292 ± 34.4 | 289 ± 45.0 | 0.49 |
| Proximal small intestine | 12.7 ± 1.48 | 12.6 ± 1.19 | 12.1 ± 1.36 | 0.19 |
| Middle small intestine | 12.2 ± 1.35 | 11.9 ± 0.95 | 11.9 ± 1.46 | 0.83 |
| Distal small intestine | 12.9 ± 1.67 | 13.2 ± 1.36 | 13.3 ± 1.68 | 0.52 |
| Stomach (empty) | 6.25 ± 0.79 | 6.65 ± 1.33 | 7.03 ± 1.97 | 0.37 |
| Colon (full) | 26.9 ± 7.85^1^ | 24.0 ± 6.51 | 25.1 ± 8.70 | 0.51 |
| Liver | 27.4 ± 2.25 | 27.4 ± 2.84 | 27.5 ± 3.41 | 0.99 |
| Spleen | 3.63 ± 0.79 | 3.18 ± 0.87 | 3.57 ± 0.62 | 0.21 |
| Heart | 7.79 ± 1.21 | 7.86 ± 2.16 | 7.62 ± 1.03 | 0.77 |
| Lungs | 22.0 ± 6.05 | 22.3 ± 5.43 | 21.7 ± 4.82 | 0.88 |
| Kidneys | 7.00 ± 1.20 | 7.76 ± 0.99 | 7.27 ± 1.33 | 0.15 |

WPC-A-EV: whey protein concentrate from acid whey enriched in extracellular vesicles; WPC-PL: whey protein concentrate enriched in phospholipids; SL: soy lecithin

^1^One missing value in the WPC-A-EV group for colon weight

**Table S3.** Absolute brain weights and brain water content on day 19 in WPC-A-EV, WPC-PL and SL groups (mean ± sd)

|  | **Absolute brain weight (g)** | | |  |
| --- | --- | --- | --- | --- |
| **Organ** | **WPC-A-EV (*n*=22)** | **WPC-PL (*n*=19)** | **SL (*n*=19)** | ***p*** |
| Total weight | 35.5±2.23 | 35.8±1.60 | 35.9±2.64 | 0.90 |
| Cerebellum | 3.76±0.33 | 3.77±0.22 | 3.82±0.23 | 0.82 |
| Cerebrum | 28.2±1.76 | 28.4±1.42 | 28.4±2.19 | 0.96 |
| Brain stem | 3.35±0.26 | 3.44±0.18 | 3.48±0.27 | 0.28 |
| Left hippocampus | 0.60±0.08 | 0.60±0.09 | 0.60±0.06 | 0.97 |
| Left caudate nucleus | 0.32±0.06 | 0.33±0.07 | 0.35±0.04 | 0.33 |
| Water percentage | 0.829±0.005 | 0.832±0.011 | 0.827±0.006 | 0.41 |

WPC-A-EV: whey protein concentrate from acid whey enriched in extracellular vesicles; WPC-PL: whey protein concentrate enriched in phospholipids; SL: soy lecithin

**Table S4.** Magnetic resonance imaging (MRI) derived measurements of mean diffusivity, fractional anisotropy and volumes of 8 brain regions in the right hemisphere of the brain in WPC-A-EV, WPC-PL and SL groups (mean ± sd)

| **Parameter** | **WPC-A-EV (*n*=22)** | | **WPC-PL (*n*=19)** | | **SL (*n*=19)** | | ***p*** |
| --- | --- | --- | --- | --- | --- | --- | --- |
| **Mean diffusivity (µm^2^/s)** |  |  | |  | |  | |
| Nucleus accumbens | 0.316±0.020 | | 0.320±0.014 | | 0.321±0.021 | | 0.83 |
| Caudate nucleus | 0.317±0.020 | | 0.326±0.018 | | 0.324±0.022 | | 0.45 |
| Lentiform nucleus | 0.317±0.015 | | 0.324±0.012 | | 0.322±0.022 | | 0.61 |
| Fornix | 0.262±0.018 | | 0.266±0.026 | | 0.259±0.020 | | 0.50 |
| Hippocampus | 0.308±0.016 | | 0.308±0.009 | | 0.321±0.017 | | <0.01 |
| Amygdala | 0.321±0.017 | | 0.315±0.016 | | 0.317±0.022 | | 0.17 |
| Prefrontal cortex | 0.325±0.019 | | 0.326±0.013 | | 0.325±0.016 | | 0.94 |
| Internal capsule | 0.265±0.011 | | 0.269±0.009 | | 0.269±0.017 | | 0.69 |
| **Fractional anisotropy** |  | |  | |  | |  |
| Nucleus accumbens | 0.148±0.027 | | 0.142±0.026 | | 0.136±0.027 | | 0.34 |
| Caudate nucleus | 0.148±0.015 | | 0.153±0.014 | | 0.148±0.012 | | 0.54 |
| Lentiform nucleus | 0.193±0.014 | | 0.189±0.013 | | 0.191±0.011 | | 0.32 |
| Fornix | 0.553±0.027 | | 0.547±0.037 | | 0.538±0.032 | | 0.23 |
| Hippocampus | 0.172±0.011 | | 0.174±0.016 | | 0.173±0.010 | | 0.87 |
| Amygdala | 0.158±0.021 | | 0.165±0.023 | | 0.156±0.017 | | 0.38 |
| Prefrontal cortex | 0.217±0.027 | | 0.219±0.026 | | 0.210±0.014 | | 0.67 |
| Internal capsule | 0.401±0.021 | | 0.412±0.025 | | 0.405±0.027 | | 0.55 |
| **Volume (mm^3^)** |  | |  | |  | |  |
| Nucleus accumbens | 37.9±3.68 | | 38.3±4.58 | | 38.2±2.67 | | 0.98 |
| Caudate nucleus | 177±17.4 | | 179±11.3 | | 181±15.9 | | 0.86 |
| Lentiform nucleus | 173±17.5 | | 176±10.3 | | 178±15.7 | | 0.47 |
| Fornix | 10.5±1.15 | | 10.4±0.86 | | 10.2±1.02 | | 0.61 |
| Hippocampus | 277±24.1 | | 287±15.6 | | 289±23.3 | | 0.31 |
| Amygdala | 45.6±6.04 | | 45.0±4.02 | | 45.0±5.22 | | 0.82 |
| Prefrontal cortex | 878±67.5 | | 894±60.6 | | 887±96.1 | | 0.84 |
| Internal capsule | 90.1±9.90 | | 89.9±8.33 | | 90.3±9.06 | | 1.00 |

WPC-A-EV: whey protein concentrate from acid whey enriched in extracellular vesicles; WPC-PL: whey protein concentrate enriched in phospholipids; SL: soy lecithin

**
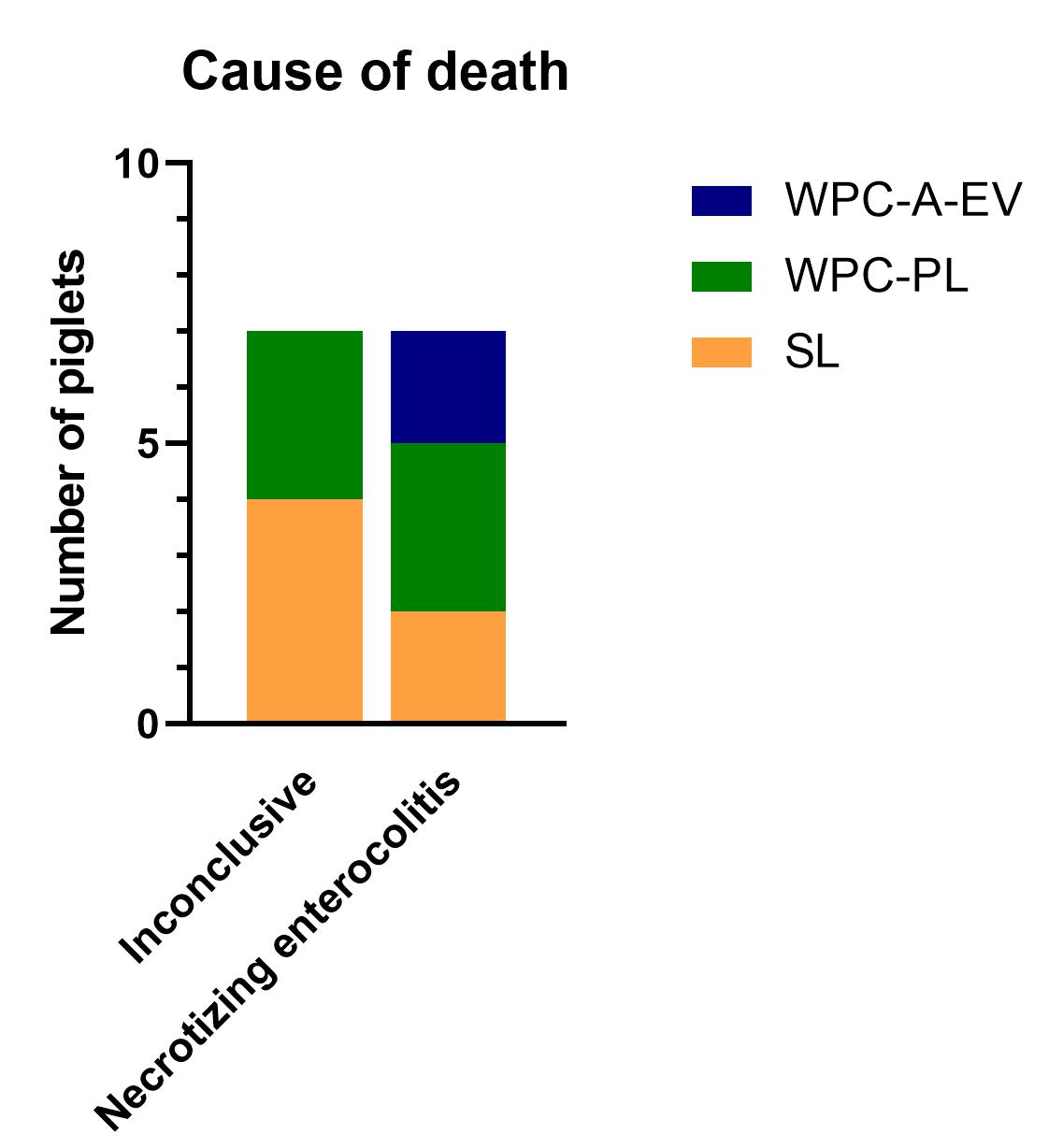
**

**Figure S1.** Cause of death in piglets euthanized during the 19-day study period as determined by necropsy in whey protein concentrate from acid whey enriched in extracellular vesicles (WPC-A-EV, blue, *n*=2), whey protein concentrate enriched in phospholipids (WPC-PL, green, *n*=6,) and soy lecithin (SL, orange, *n*=6) groups. Inconclusive = no clear cause of death.

**Figure S2.** Growth curves over 19 days for preterm piglets fed a milk formula diet containing whey protein concentrate from acid whey enriched in extracellular vesicles (WPC-A-EV, blue, *n*=22), whey protein concentrate enriched in phospholipids (WPC-PL, green, *n*=19) and soy lecithin (SL, orange, *n*=19) as the emulsifier. Data are presented as mean ± sd.

**Figure S3.** Buffer capacity of whey protein concentrate from acid whey enriched in extracellular vesicles (WPC-A-EV), whey protein concentrate enriched in phospholipids (WPC-PL) or soy lecithin (SL) emulsions (filled columns, *n*=3) and their corresponding intact diets (outlined columns, *n*=3). Data are presented as mean ± sd. ***p<0.001.


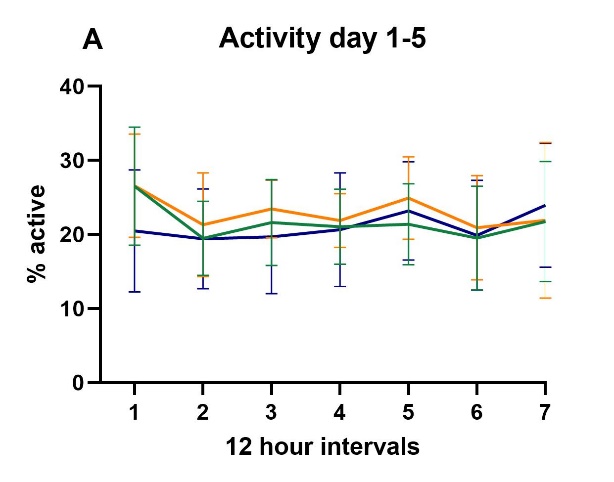

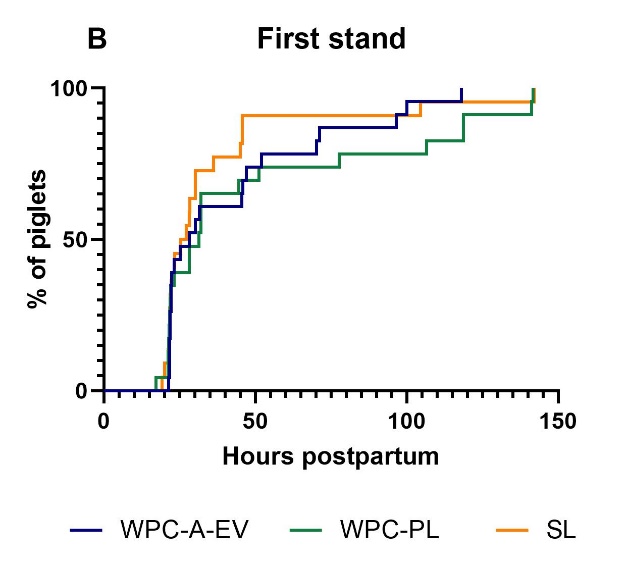


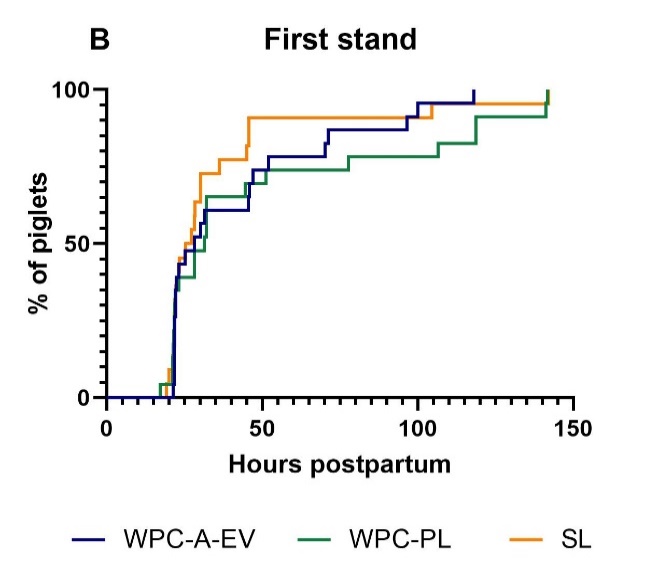

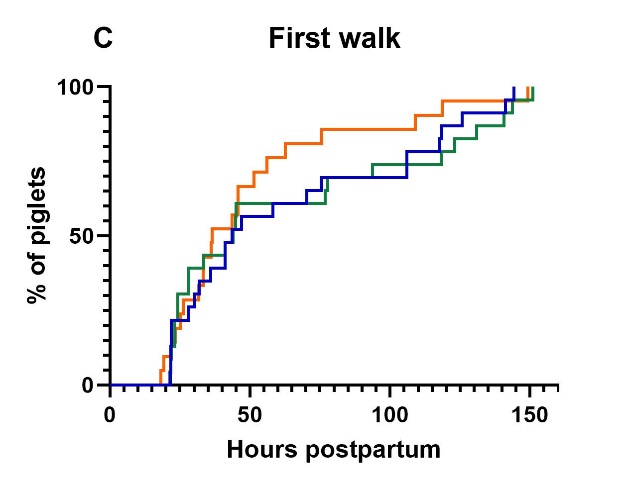

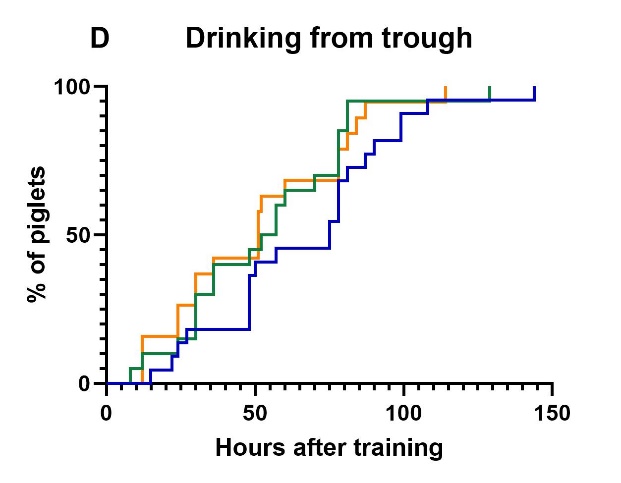


**Figure S4.** Home cage activity and basic motor skill acquisition. **(A)** Percentage of active time during postnatal day 2-5. Time before a given percentage of piglets had their first **(B)** stand **(C)** walk and **(D)** full milk bolus drink from a trough. WPC-A-EV: whey protein concentrate from acid whey enriched in extracellular vesicles (blue, activity: *n*= 12-19, stand: *n*=23, walk: *n*=23, drink: *n*=22), WPC-PL: whey protein concentrate enriched in phospholipids (green, activity: *n*=14-22, stand: *n*=22, walk: *n*=22, drink: *n*=20), SL: soy lecithin (orange, activity: *n*=10-19, stand: *n*=22, walk: *n*=21, drink: *n*=19).


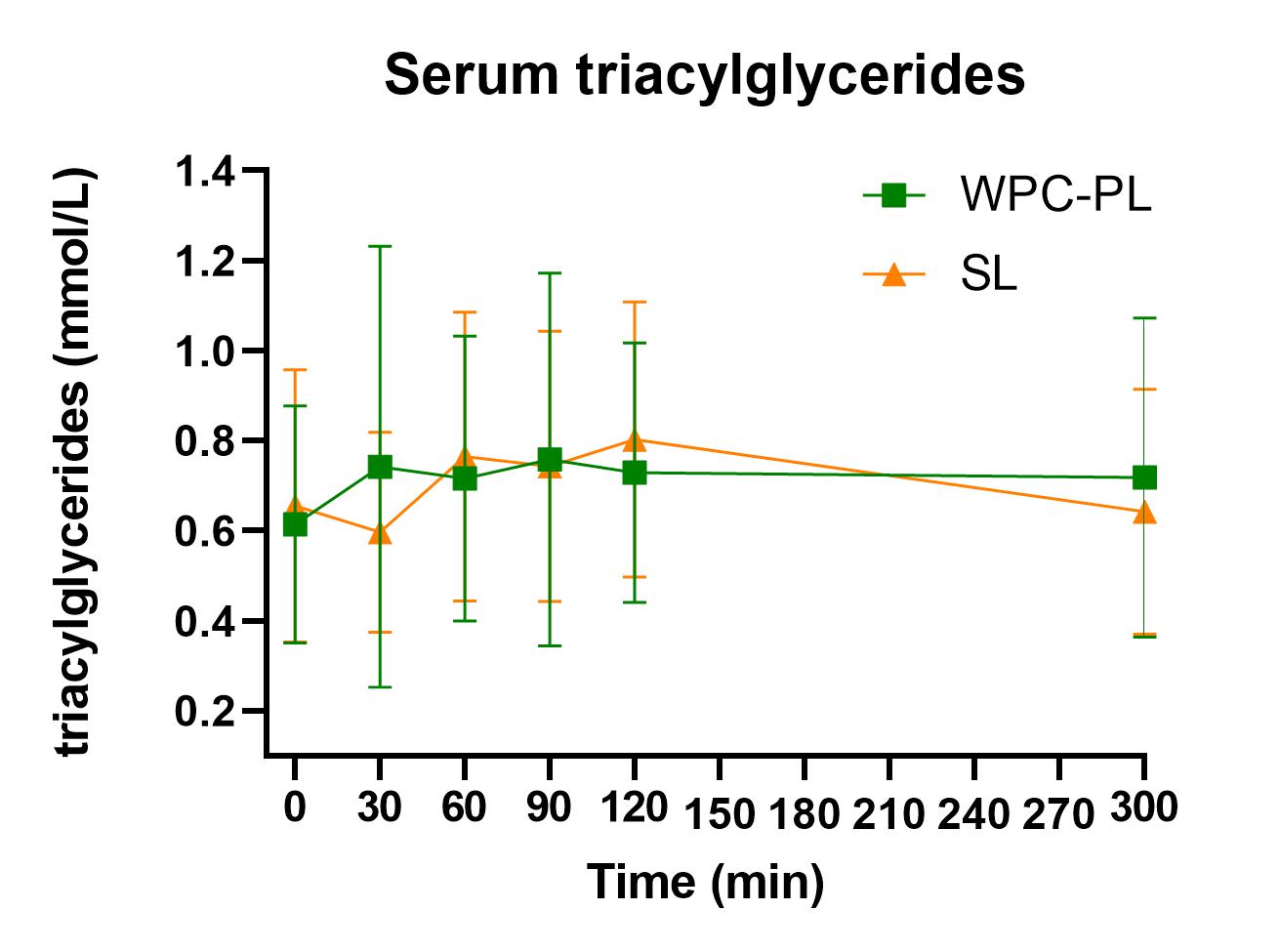


**Figure S5.** Serum triacylglyceride levels at 0, 30, 60, 90, 120 and 300 min after a 20 ml/kg bolus of the intact whey protein concentrate enriched in phospholipids (WPC-PL, green) or soy lecithin (SL, orange) diet in a follow-up experiment. Data are presented as mean ± sd.
